# Monocyte Distribution Width as a Biomarker for Predicting Bacteremia: A Retrospective Study in the Emergency Department

**DOI:** 10.3390/life16010178

**Published:** 2026-01-22

**Authors:** Tse-Hao Chen, Yu-Jang Su, Wei-Hsiang Liao, Weide Tsai, Ding-Kuo Chien, Wen-Han Chang, Chyi-Huey Bai

**Affiliations:** 1 Department of Emergency Medicine, MacKay Memorial Hospital, Taipei 104217, Taiwan; chenth1020@gmail.com (T.-H.C.); pioneermd1@gmail.com (Y.-J.S.); a121980121@gmail.com (W.-H.L.); mmhededucation@gmail.com (W.T.); anderson1021dr@gmail.com (D.-K.C.); branden888@gmail.com (W.-H.C.); 2 Toxicology Division, Department of Emergency Medicine, MacKay Memorial Hospital, Taipei 104217, Taiwan; 3 Graduate Institute of Injury Prevention and Control, College of Public Health, Taipei Medical University, Taipei 110313, Taiwan; 4 Department of Public Health, School of Medicine, Taipei Medical University, Taipei 110313, Taiwan; 5 Department of Medicine, MacKay Medical College, New Taipei City 25245, Taiwan; 6 MacKay Junior College of Medicine, Nursing, and Management, New Taipei City 11260, Taiwan; 7 School of Public Health, College of Public Health, Taipei Medical University, Taipei 110313, Taiwan; 8 Nutrition Research Center, Taipei Medical University Hospital, Taipei 110313, Taiwan

**Keywords:** monocyte distribution width, bacteremia, emergency department, diagnostic accuracy

## Abstract

Blood culture is the diagnostic gold standard for bacteremia in the emergency department (ED), but its turnaround time can delay appropriate antimicrobial therapy, highlighting the need for rapid, accessible biomarkers. We retrospectively analyzed adult ED patients from July 2023 to June 2024 who underwent blood culture testing and had complete data for monocyte distribution width (MDW), white blood cell count (WBC), C-reactive protein (CRP), and neutrophil-to-lymphocyte ratio (NLR). Discrimination was assessed using area under the receiver operating characteristic curve (AUROC) and diagnostic accuracy using sensitivity, specificity, and diagnostic odds ratio (DOR); combined models were compared with net reclassification improvement (NRI) and integrated discrimination improvement (IDI). Among 19,325 patients, 2011 (10.4%) had positive blood cultures. MDW had the highest AUROC (0.760) versus CRP (0.730), NLR (0.695), and WBC (0.642); at a cut-off of 22, MDW showed 0.72 sensitivity, 0.68 specificity, and DOR 5.46. The best combined model was MDW+NLR (AUROC 0.785; DOR 6.39; NRI 0.428; IDI 0.770). MDW is a rapid and effective marker for identifying bacteremia in the ED, and performance improves when combined with NLR.

## 1. Introduction

Sepsis is a life-threatening organ dysfunction resulting from dysregulated host response to infection and remains one of the leading causes of mortality worldwide [1]. Global Burden of Disease estimates indicate that sepsis remains a major cause of morbidity and mortality worldwide, with 48.9 million cases and 11.0 million deaths in 2017 and a marked increase to 166 million cases and 21.4 million sepsis-related deaths in 2021 [2,3]. Among the various infectious etiologies of sepsis, bloodstream infections, particularly those presenting as bacteremia, are of particular concern due to their capacity to rapidly disseminate pathogens and trigger systemic inflammatory responses [4,5]. Currently, blood culture remains the gold standard for diagnosing bacteremia [4,5]. However, actionable microbiologic information often becomes available only after culture incubation and laboratory workflow, with Gram stain reporting typically occurring on the order of about a day after specimen collection and definitive organism identification and susceptibility results requiring additional time [6,7]. Such diagnostic delays can postpone initiation of appropriate antimicrobial therapy and contribute to increased mortality. Moreover, previous studies have reported that up to 16.6% of patients are recalled after hospital discharge due to newly identified positive blood cultures or the need for treatment modification [8].

Monocyte distribution width (MDW) is a novel hematologic parameter that quantifies variability in monocyte cell volume, which increases in response to systemic inflammation or infection. During the early stages of immune activation, such as in sepsis or other inflammatory conditions, monocytes undergo morphological changes that lead to a broader cell size distribution. This feature can be detected by specific automated hematology analyzers [9]. Importantly, MDW is calculated from routine complete blood count (CBC) with differential, requiring no additional blood draw or processing time, thereby, offering a rapid, cost-effective and widely available alternative. On the other hand, other biomarkers for early detection of sepsis or bacteremia may have longer turnaround times, require additional blood sampling, or incur additional costs, which may limit their routine use in the emergency department. These characteristics make MDW a practical and noninvasive biomarker for early identification of immune activation in emergency settings [9].

MDW has been increasingly recognized as a valuable tool for early identification of sepsis, but its utility in predicting bacteremia remains underexplored [10,11]. Given the clinical urgency of promptly identifying bloodstream infections, this study aimed to evaluate the discriminative performance of MDW in detecting bacteremia, as confirmed by positive blood culture, at the emergency department (ED).

## 2. Materials and Methods

### 2.1. Study Design

This retrospective observational study was conducted at the ED of a tertiary care hospital in Taiwan. The study adhered to the Transparent Reporting of a Multivariable Prediction Model for Individual Prognosis or Diagnosis guidelines [12].

### 2.2. Study Population

The study population comprised adult patients (aged ≥ 18 years) who were seen at the ED between July 2023 and June 2024. All patients who were clinically suspected of having an infectious disease and had blood cultures obtained during their ED visit were consecutively included during the study period. Clinical suspicion of infection was typically accompanied by documented signs suggestive of infection or sepsis, such as fever, qSOFA-related physiological abnormalities (respiratory rate ≥ 22/min, systolic blood pressure ≤ 100 mmHg, or altered mental status), or both. The following exclusion criteria were applied: (1) age < 18 years; (2) absence of blood culture results; (3) missing laboratory data for MDW, white blood cell (WBC) count, C-reactive protein (CRP), or neutrophil-to-lymphocyte ratio (NLR); and (4) incomplete demographic data.

### 2.3. Ethics Statement

The study protocol was reviewed and approved by the Institutional Review Board of MacKay Memorial Hospital (approval no. 24MMHIS386e). The requirement for informed consent was waived due to the retrospective design of the study and the use of de-identified clinical data.

### 2.4. Variables

The predictors evaluated in the area under the receiver operating characteristic curve (AUROC) analysis were MDW, WBC, CRP, and NLR, and the reference outcome was blood culture positivity. Laboratory tests were ordered at the discretion of ED physicians as part of routine care and were obtained within the first 2 h of ED presentation, in accordance with standard clinical practice. Baseline patient characteristics included age, sex and medical history of hypertension, diabetes mellitus, cardiovascular disease, pulmonary disease, liver disease, cerebrovascular accident, neoplasm, and chronic kidney disease. The initial vital signs recorded upon ED arrival were also collected.

CBC parameters, including MDW and WBC, neutrophil and lymphocyte counts, were measured using a hematology analyzer (DxH 900; Beckman Coulter, Brea, CA, USA) [9]. MDW was automatically calculated as the standard deviation of the monocyte cell volume within the CBC. NLR was derived by dividing the absolute neutrophil count by the absolute lymphocyte count. CRP levels were measured using a chemistry analyzer (DxC 700 AU; Beckman Coulter).

Blood cultures were performed using the BACTEC FX blood culture system (Becton Dickinson, Franklin Lakes, NJ, USA). Each culture set comprised two bottles: one aerobic and one anaerobic. Blood culture was defined as positive if at least one bottle demonstrated microbial growth, excluding common skin contaminants, such as coagulase-negative staphylococci, *Propionibacterium acnes*, *Corynebacterium* spp., and *Bacillus* spp.

### 2.5. Outcomes

The primary outcome of this study was the predictive performance of MDW, WBC, CRP, and NLR in identifying positive blood culture results. The secondary outcome was comparison of the diagnostic performance of MDW in combination with WBC, CRP, or NLR in predicting bacteremia.

### 2.6. Statistical Analyses

Continuous variables were compared using the independent-samples t-test or the Mann–Whitney U test, as appropriate. Given the right-skewed distributions of MDW, WBC, CRP, and NLR, group comparisons for these biomarkers were performed using the Mann–Whitney U test. Categorical variables were analyzed using the Pearson χ^2^ or Fisher’s exact test, depending on the sample size. A two-sided *p* value of <0.05 was considered statistically significant.

The diagnostic performance of each biomarker was assessed by calculating the AUROC, sensitivity, specificity, positive predictive value (PPV), negative predictive value (NPV), and diagnostic odds ratio (DOR), along with their corresponding 95% confidence intervals (CIs). AUROCs with 95% CIs were estimated by the DeLong method, and pairwise AUROC comparisons were performed using DeLong’s test [13]. The optimal cut-off value for each biomarker was determined using Youden’s index [14]. To evaluate whether combining two biomarkers improved predictive performance, the net reclassification index (NRI) and integrated discrimination index (IDI) were calculated [15]. In addition, combined biomarker models (MDW + CRP, MDW + WBC, and MDW + NLR) were constructed using multivariable logistic regression, and the predicted probabilities from each model were used for AUROC analyses. Subgroup analyses were performed based on age (elderly, ≥65 years vs. non-elderly, <65 years); sex (men vs. women) and type of bacteremia (Gram-positive vs. Gram-negative). All analyses were performed using R software, version 4.4.2 [16].

## 3. Results

**Figure 1:**
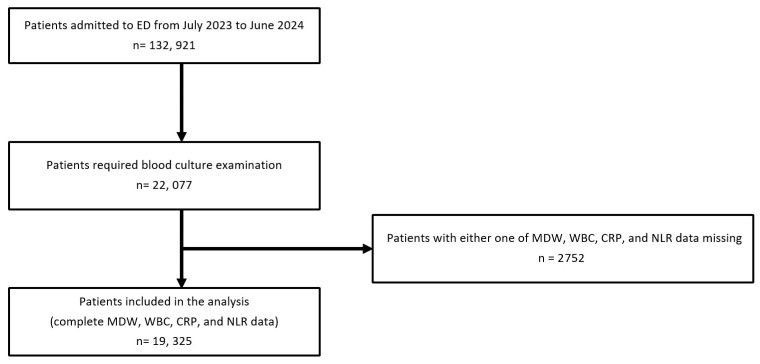
Study flowchart for patient inclusion in the bacteremia prediction analysis. ED, emergency department; MDW, monocyte distribution width; WBC, white blood cell count; CRP, C-reactive protein; NLR, neutrophil-to-lymphocyte ratio.

Of the 132,921 patients during the study period, 22,077 underwent blood culture examinations. After excluding 2752 patients because of missing data on MDW, WBC, CRP, and/or NLR, 19,325 patients with complete biomarker data were included in the final analysis. The flow diagram outlining the inclusion and exclusion process is shown in Figure 1. We also compared baseline characteristics between included and excluded patients to evaluate potential selection related to missing biomarker data (Supplementary Table S1).

The baseline characteristics of patients with and without bacteremia are summarized in Table 1. The overall mean age of the study cohort was 63.86 ± 20.15 years, and 51% were men. Of 19,325 patients, 2011 (10.4%) had positive blood cultures. The distribution of isolated pathogens among positive blood cultures is provided in Supplementary Table S2. Compared with the non-bacteremia group, the bacteremia group was significantly older and had a higher prevalence of hypertension, diabetes mellitus, cardiovascular disease, liver disease, cerebrovascular accident, and neoplastic disease; had significantly higher body temperature, heart rate and respiratory rate and lower oxygen saturation and Glasgow Coma Scale scores upon ED presentation; and had significantly higher levels of all four predictive biomarkers: MDW, CRP, NLR, and WBC. Results of the adjusted multivariable models are provided in Supplementary Table S3.

**Figure 2:**
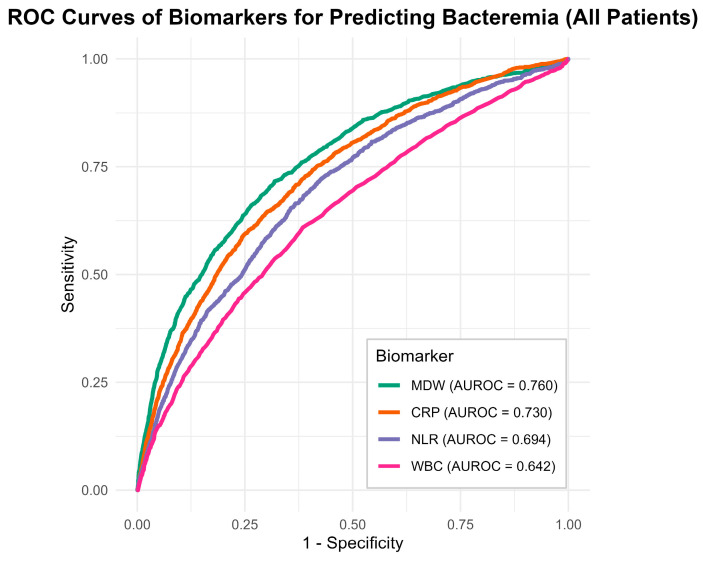
Comparison of diagnostic performance of MDW, CRP, NLR, and WBC in predicting bacteremia, evaluated by area under the ROC curve (AUROC).

The diagnostic performance of each biomarker for predicting bacteremia is presented in Figure 2 and Table 2. Among the four biomarkers, predictive value was significantly the highest for MDW (AUROC 0.760, 95% CI 0.748–0.774), followed by CRP (AUROC 0.730, 95% CI 0.717–0.743); NLR (AUROC 0.695, 95% CI 0.680–0.708); and WBC (AUROC 0.642, 95% CI 0.626–0.655) (*p* < 0.01 for all). The optimal MDW cut-off value for predicting positive blood cultures was 22, with sensitivity of 0.72, specificity of 0.68, PPV of 20.72%, and NPV of 95.44%. The DOR for MDW was 5.46, outperforming CRP (DOR 4.22), NLR (DOR 3.42), and WBC (DOR 2.49). Post hoc comparisons confirmed the ranking of diagnostic performance as MDW > CRP > NLR > WBC.

The diagnostic performance of each biomarker in the subgroup analyses is presented in Table 3. Across all subgroups, MDW showed consistently favorable diagnostic performance. Additionally, subgroup analyses by primary infection site showed that MDW consistently outperformed NLR and WBC, and generally outperformed CRP except in soft tissue infections where MDW and CRP performed comparably. Notably, in the Gram-negative bacteremia, elderly, and men subgroups, MDW outperformed CRP in terms of AUROC and DOR. On the other hand, in the Gram-positive bacteremia, non-elderly, and women subgroups, MDW and CRP showed no significant differences in diagnostic performance.

**Figure 3:**
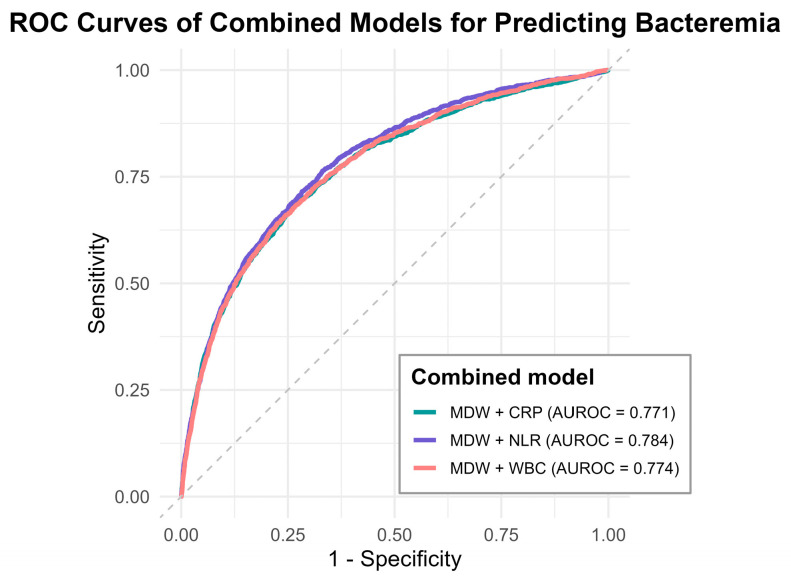
Evaluation of combined models using MDW with CRP, NLR, or WBC, demonstrating improved predictive accuracy compared to individual biomarkers.

To evaluate whether combining MDW with other biomarkers could improve bacteremia prediction, we constructed three two-biomarker models: MDW + NLR, MDW + WBC and MDW + CRP. These combined models were fitted using multivariable logistic regression, and the predicted probabilities from the fitted models were used for AUROC analyses; the model coefficients (ORs with 95% CIs) and final equations are provided in Supplementary Table S4. As shown in Figure 3 and Table 4, diagnostic performance was the highest for MDW + NLR (AUROC 0.785, 95% CI 0.773–0.797), followed by MDW + WBC (AUROC 0.774, 95% CI 0.762–0.787) and MDW + CRP (AUROC 0.772, 95% CI 0.759–0.785). Moreover, the MDW + NLR model demonstrated the highest DOR (6.387), with favorable NRI (0.428) and IDI (0.770). All comparisons were statistically significant (*p* < 0.05). Post hoc analysis further indicated that MDW + NLR performed significantly better than the other two combinations, whereas the performances of MDW + WBC and MDW + CRP were comparable.

## 4. Discussion

Our findings demonstrated that MDW outperformed traditional inflammatory biomarkers, such as WBC, CRP and NLR, in predicting bacteremia among patients at the ED. We identified an optimal MDW cut-off value of 22, which achieved a DOR > 5, providing a practical threshold beyond the manufacturer’s recommended cut-off of 20 for MDW (Beckman Coulter). Furthermore, we found that combining MDW with NLR yielded additional discriminatory power and is a potentially useful dual-marker model for early bacteremia detection.

To the best of our knowledge, this was the first formal study to evaluate the utility of MDW in predicting bacteremia. Previous studies primarily focused on the role of MDW in early sepsis detection, whereas our study specifically targeted bloodstream infections, which represent a distinct clinical entity that requires prompt intravenous antibiotic therapy. In addition, our study included one of the largest single-center cohorts reported to date. These findings provided robust real-world evidence supporting the incorporation of MDW into routine ED screening for early bacteremia detection.

Prior to this study, the usefulness of MDW in detecting sepsis has already been established [11,17,18,19]. However, its application in predicting bloodstream infections has not been widely explored. To date, the only report addressing this topic appears to be a conference abstract [20]. In that study, MDW at a cut-off value of 20 demonstrated 83.9% sensitivity and 36.2% specificity; the reported NPV was 93.0%, but the DOR was approximately 3.0, indicating that an MDW of 20 may be insufficient for clinical use as a stand-alone predictor. Moreover, that study included a smaller sample size of 5316 patients and did not provide a head-to-head comparison with other biomarkers. On the other hand, our study included a substantially larger cohort and systematically compared MDW with other commonly used inflammatory markers. These enhancements led to more robust evidence on the value of MDW in predicting bacteremia in emergency settings. Notably, MDW showed better discrimination in Gram-negative than Gram-positive bacteremia, which may reflect differences in early innate immune activation. Gram-negative pathogens via lipopolysaccharide-mediated signaling often trigger a more rapid and pronounced systemic inflammatory response than Gram-positive cell-wall components such as peptidoglycan and lipoteichoic acid, potentially resulting in less prominent early monocyte morphological changes captured by MDW in Gram-positive infections [21]. Despite this difference, MDW still performed comparably to CRP and outperformed NLR and WBC for predicting Gram-positive bacteremia in our cohort.

In this study, the optimal MDW cut-off value for predicting bacteremia was 22, which is higher than the manufacturer’s recommended cut-off of 20 (Beckman Coulter) and the cut-off values reported in previous studies [9,10,11,17,18]. The discrepancy likely stems from the different clinical endpoints among the studies. Most previous researched focused on early detection of sepsis, which encompasses a broad clinical spectrum and may be managed with either oral or intravenous antibiotics [22,23,24]. On the other hand, our study specifically targeted bacteremia, which represents a more narrowly defined and clinically significant subset of infection that typically requires immediate intravenous antibiotic therapy. In practice, MDW cut-offs should be selected according to the intended use. For rule-in or escalation decisions aimed at identifying patients at higher risk of bacteremia, our findings support using a higher threshold such as MDW ≥ 22. MDW should be interpreted together with clinical assessment and other laboratory or imaging findings. Notably, a previous study that examined the relationship between MDW and time to antibiotic administration reported a cut-off value of 21.5 [19], which is similar to our clinical decision threshold. These findings support the notion that a higher MDW cut-off may be more appropriate in guiding treatment decisions for patients who are more likely to require intravenous antibiotics.

MDW has emerged as a promising and rapidly available adjunct for early risk stratification in ED settings, particularly during the interval while awaiting blood culture results. In our cohort, the negative predictive value of 95.44% suggests that MDW may help identify patients at relatively lower risk of bacteremia. MDW combined with NLR might be a simple and effective prediction model, considering that both biomarkers are routinely obtained through CBC and can be determined within 10 min. Given its fast turnaround time, accessibility, and integration potential, this model could be readily incorporated into electronic health record systems to support real-time clinical decision-making at the ED, where timely intervention is critical to patient outcomes.

This study had several limitations. First, it used a retrospective design, which inherently increases the risk of incomplete or missing data. We compared included and excluded patients (Supplementary Table S1) and found differences suggesting that biomarker testing may have been influenced by clinical decision-making. Nevertheless, the large sample size, which is the largest reported to date, provided robust real-world evidence supporting the predictive value of MDW for bacteremia. Second, we did not include certain biomarkers, such as procalcitonin and lactate, for comparison. However, in clinical practice, the use of these biomarkers may be limited by selection bias and cost considerations, particularly in healthcare systems with constrained medical budget. On the other hand, MDW offers a rapid and cost-effective alternative that can be obtained from routine CBC without additional expense. Third, the optimal MDW cut-off value of 22 that we identified in this large single-center cohort requires external validation. Furthermore, we did not develop or validate an integrated prediction model that combines MDW with bedside clinical parameters such as vital signs or severity scores and with other laboratory biomarkers, which may yield improved discrimination for bacteremia. Future multi-center prospective studies are warranted to confirm the generalizability and clinical utility of our findings.

## 5. Conclusions

Among patients at the ED, an MDW of 22 outperformed traditional markers and can be a valuable biomarker with strong diagnostic performance for early identification of bacteremia. Combining MDW with NLR further enhanced predictive accuracy and has the potential use as a rapid and practical model for early bacteremia screening.

## Figures and Tables

**Table 1:** Baseline characteristics of patients with and without bacteremia.

	Total (n = 19,325)	Bacteremia (n = 2011)	Non-Bacteremia (n = 17,314)	*p* Value
Patient characteristics				
Age	63.86 ± 20.15	69.23 ± 16.64	63.34 ± 20.29	<0.05
Male	51%	51%	51%	0.95
Hypertension	33.94%	39.92%	33.36%	<0.05
DM	24.36%	30.92%	23.73%	<0.05
CVD	15.56%	17.80%	15.35%	<0.05
Pulmonary disease	1.00%	0.59%	1.04%	0.051
Liver disease	0.98%	1.62%	0.92%	<0.05
Stroke	5.31%	7.96%	5.05%	<0.05
Neoplasm	0.68%	0.69%	0.68%	<0.05
CKD	3.88%	4.23%	3.85%	0.393
Initial vital signs at emergency department				
Body temperature (°C)	37.27 ± 0.82	37.94 ± 1.51	37.21 ± 1.13	<0.05
Heart rate (bpm)	99.81 ± 18.27	107.42 ± 25.12	99.09 ± 22.99	<0.05
Respiratory rate	19.92 ± 1.74	20.10 ± 3.75	19.90 ± 3.21	<0.05
SBP (mmHg)	133.25 ± 26.08	135.46 ± 28.15	132.98 ± 24.66	<0.05
DBP (mmHg)	95.77 ± 18.54	73.84 ± 17.51	76.40 ± 15.33	<0.05
SpO_2_ (%)	96.35 ± 4.95	95.79 ± 5.71	96.41 ± 5.24	<0.05
GCS	14.02 ± 1.40	13.42 ± 3.21	14.08 ± 2.55	<0.05
qSOFA ≥ 2	38.37%	73%	43%	<0.05
Prediction biomarkers for bacteremia				
MDW	21.39 ± 5.33	26.64 ± 7.37	20.93 ± 4.85	<0.05
NLR	10.34 ± 11.49	18.40 ± 18.66	9.66 ± 10.74	<0.05
WBC (10^3^/μL)	11.85 ± 6.94	13.94 ± 7.65	11.53 ± 8.80	<0.05
CRP (mg/dL)	7.12 ± 8.22	13.67 ± 10.74	6.58 ± 7.80	<0.05

Data are presented as mean ± standard deviation (SD) or percentage (%), as appropriate. Because MDW, WBC, CRP, and NLR were right-skewed with extreme values, between-group comparisons for these biomarkers were performed using the Mann–Whitney U test. Abbreviation: DM: diabetes mellitus; CVD: cardiovascular disease; CKD: chronic kidney disease; SpO_2_: peripheral oxygen saturation; GCS: Glasgow Coma Scale; MDW: monocyte distribution width; NLR: neutrophil-to-lymphocyte ratio; WBC: white blood cell count; CRP: C-reactive protein; bpm: beats per minute.

**Table 2:** Diagnostic performance of individual biomarkers for predicting bacteremia.

Biomarker	AUROC (95% CI)	Optimal Cut-Off Value	Sensitivity	Specificity	PPV	NPV	DOR	Post Hoc Pairwise Analysis
MDW	0.76 (0.75–0.77)	22.01	0.72	0.68	20.72%	95.44%	5.46	MDW > CRP > NLR > WBC
CRP	0.73 (0.72–0.74)	8.48	0.65	0.70	20.11%	94.51%	4.22	
NLR	0.70 (0.68–0.71)	8.96	0.65	0.64	17.34%	94.03%	3.42	
WBC	0.64 (0.63–0.66)	11.95	0.61	0.62	15.71%	93.19%	2.49	

Abbreviations: AUROC: area under the receiver operating characteristic curve; CI: confidence interval; MDW: monocyte distribution width; CRP: C-reactive protein; NLR: neutrophil-to-lymphocyte ratio; WBC: white blood cell count; PPV: positive predictive value; NPV: negative predictive value; DOR: diagnostic odds ratio. Post hoc pairwise AUROC comparisons were performed using the DeLong test.

**Table 3:** Subgroup analysis of biomarker performance for predicting bacteremia across pathogen types, age and sex.

	Biomarker	AUROC (95% CI)	Sensitivity	Specificity	DOR	Post Hoc Pairwise Analysis
All patients N = 19,325	MDW	0.76 (0.75–0.77)	0.72	0.68	5.46	MDW > CRP > NLR > WBC
	CRP	0.73 (0.72–0.74)	0.65	0.70	4.33	
	NLR	0.69 (0.68–0.71)	0.65	0.64	3.30	
	WBC	0.64 (0.63–0.66)	0.61	0.62	2.55	
GNB N = 1066	MDW	0.79 (0.77–0.80)	0.71	0.74	6.97	MDW > CRP > NLR > WBC
	CRP	0.74 (0.73–0.76)	0.62	0.75	4.89	
	NLR	0.71 (0.69–0.72)	0.72	0.59	3.70	
	WBC	0.64 (0.62–0.66)	0.61	0.61	2.45	
GPB N = 560	MDW	0.69 (0.67–0.71)	0.61	0.66	3.04	MDW = CRP > NLR = WBC
	CRP	0.69 (0.67–0.71)	0.60	0.68	3.19	
	NLR	0.66 (0.63–0.68)	0.63	0.62	2.78	
	WBC	0.64 (0.61–0.66)	0.62	0.60	2.45	
Elderly N = 10,644	MDW	0.76 (0.74–0.77)	0.67	0.72	5.22	MDW > CRP > NLR > WBC
	CRP	0.72 (0.70–0.73)	0.70	0.62	3.81	
	NLR	0.69 (0.67–0.71)	0.65	0.63	3.16	
	WBC	0.65 (0.63–0.67)	0.60	0.65	2.79	
Non-Elderly N = 8681	MDW	0.76 (0.73–0.78)	0.71	0.70	5.71	MDW = CRP > NLR > WBC
	CRP	0.75 (0.73–0.77)	0.63	0.77	5.70	
	NLR	0.70 (0.68–0.72)	0.72	0.60	3.86	
	WBC	0.64 (0.61–0.66)	0.58	0.65	2.56	
Male N = 9862	MDW	0.75 (0.74–0.77)	0.69	0.70	5.19	MDW > CRP = NLR > WBC
	CRP	0.71 (0.69–0.73)	0.63	0.70	3.97	
	NLR	0.69 (0.67–0.71)	0.67	0.62	3.31	
	WBC	0.62 (0.60–0.64)	0.58	0.62	2.25	
Female N = 9463	MDW	0.77 (0.75–0.79)	0.68	0.73	5.75	MDW = CRP > NLR > WBC
	CRP	0.75 (0.73–0.77)	0.65	0.72	4.78	
	NLR	0.70 (0.68–0.72)	0.73	0.59	3.89	
	WBC	0.67 (0.65–0.69)	0.64	0.62	2.90	
Respiratory N = 5752	MDW	0.73 (0.71–0.76)	0.57	0.8	5.44	MDW > CRP > NLR > WBC
	CRP	0.69 (0.66–0.72)	0.61	0.69	3.49	
	NLR	0.64 (0.61–0.68)	0.57	0.67	2.64	
	WBC	0.60 (0.57–0.63)	0.6	0.59	2.15	
IAI N = 4715	MDW	0.76 (0.73–0.78)	0.68	0.72	5.45	MDW > CRP = NLR > WBC
	CRP	0.70 (0.68–0.73)	0.7	0.63	4	
	NLR	0.70 (0.67–0.72)	0.65	0.65	3.42	
	WBC	0.61 (0.58–0.64)	0.61	0.56	2	
GU tract N = 5427	MDW	0.76 (0.75–0.78)	0.72	0.68	5.46	MDW > CRP > NLR > WBC
	CRP	0.73 (0.71–0.75)	0.66	0.69	4.32	
	NLR	0.69 (0.67–0.71)	0.69	0.61	3.38	
	WBC	0.64 (0.62–0.67)	0.65	0.58	2.56	
Soft tissue N = 1842	MDW	0.77 (0.73–0.80)	0.7	0.73	6.11	MDW = CRP = NLR > WBC
	CRP	0.74 (0.69–0.78)	0.72	0.66	4.95	
	NLR	0.71 (0.67–0.75)	0.71	0.64	4.31	
	WBC	0.68 (0.64–0.72)	0.64	0.65	3.36	

Abbreviations: AUROC, area under the receiver operating characteristic curve; CI, confidence interval; CRP, C-reactive protein; DOR, diagnostic odds ratio; GNB, Gram-negative bacteremia; GPB, Gram-positive bacteremia; IAI, intra-abdominal infection; MDW, monocyte distribution width; NLR, neutrophil-to-lymphocyte ratio; WBC, white blood cell count. Post hoc AUC comparisons were performed using the DeLong test.

**Table 4:** Diagnostic performance of biomarker models combining MDW with CRP, WBC, or NLR for predicting bacteremia.

Model	AUROC (95% CI)	Sensitivity	Specificity	DOR	NRI	*p* Value (NRI)	IDI	*p* Value (IDI)	Post Hoc Pairwise Analysis
MDW + NLR	0.79 (0.77–0.80)	0.72	0.72	6.39	0.43	<0.05	0.77	<0.05	MDW + NLR > MDW + WBC = MDW + CRP
MDW + WBC	0.77 (0.76–0.79)	0.64	0.78	6.16	0.31	<0.05	0.62	<0.05	
MDW + CRP	0.77 (0.76–0.79)	0.69	0.74	6.09	0.45	<0.05	0.72	<0.05	

Abbreviations: AUROC: area under the receiver operating characteristic curve; CI: confidence interval; MDW: monocyte distribution width; CRP: C-reactive protein; NLR: neutrophil-to-lymphocyte ratio; WBC: white blood cell count; NRI, net reclassification improvement; IDI, integrated discrimination improvement; DOR: diagnostic odds ratio. NRI and IDI analyses compare each combined model to MDW alone.

## Data Availability

The data are not publicly available due to restrictions regarding the Ethical Committee Institution.

## Supplementary Materials

The following supporting information can be downloaded at https://www.mdpi.com/article/10.3390/life16010178/s1, Table S1. Comparison of demographics, comorbidities, vital signs, and laboratory findings between included and excluded patients; Table S2. Most frequently isolated Gram-negative and Gram-positive pathogens from blood cultures; Table S3. Multivariable logistic regression analysis for bacteremia adjusted for medical history, initial vital signs, and laboratory biomarkers; Table S4. Logistic regression coefficients (β)/odds ratios (OR) with 95% CIs and final equations for the two-biomarker models.

## Abbreviations

The following abbreviations are used in this manuscript:

AUROC	Area Under the Receiver Operating Characteristic Curve
CBC	Complete Blood Count
CI	Confidence Interval
CKD	Chronic Kidney Disease
CRP	C-reactive Protein
CVD	Cardiovascular Disease
DM	Diabetes Mellitus
DOR	Diagnostic Odds Ratio
ED	Emergency Department
GCS	Glasgow Coma Scale
GNB	Gram-negative Bacteremia
GPB	Gram-positive Bacteremia
HTN	Hypertension
IDI	Integrated Discrimination Improvement
MDW	Monocyte Distribution Width
NLR	Neutrophil-to-Lymphocyte Ratio
NPV	Negative Predictive Value
NRI	Net Reclassification Improvement
PPV	Positive Predictive Value
SD	Standard Deviation
SPO_2_	Peripheral Oxygen Saturation
WBC	White Blood Cell Count
BPM	Beats Per Minute
