# Supplementary material for: Monocyte Distribution Width as a Biomarker for Predicting Bacteremia: A Retrospective Study in the Emergency Department
Source: Life (Basel). 2026 Jan 22;16(1):178. doi: 10.3390/life16010178 (PMC12843279; doi:10.3390/life16010178)
Supplement: Supplementary file 1 [file life-16-00178-s001.zip › life-4075133-supplementary.pdf]

Supplementary Materials

Table S1. Comparison of demographics, comorbidities, vital signs, and laboratory findings between included and excluded patients

	Included (n=19,325)	Excluded (n=2,752)	p value
Age	63.86 ± 20.15	62.67 ± 20.19	<0.05
Male	51.07%	52.31%	0.62
Hypertension	33.94%	33.03%	0.47
DM	24.36%	24.43%	0.95
CVD	15.56%	15.17%	0.68
Pulmonary disease	1.00%	1.03%	0.92
Liver disease	0.98%	1.41%	0.1
Stroke	5.31%	3.92%	<0.05
Neoplasm	0.68%	0.63%	0.87
CKD	3.88%	3.17%	0.16
Body temperature (°C)	37.27 ± 0.82	36.90 ± 1.04	<0.05
Heart rate (bpm)	99.81 ± 18.27	95.90 ± 24.92	<0.05
Respiratory rate	19.92 ± 1.74	19.38 ± 3.24	<0.05
SBP (mmHg)	133.25 ± 26.08	132.65 ± 5.33	<0.05
DBP (mmHg)	95.77 ± 18.54	96.80 ± 3.09	<0.05
SpO_2 (%)	96.35 ± 4.95	96.74 ± 4.83	<0.05
GCS	14.02 ± 1.40	14.13 ± 1.20	<0.05
qSOFA	38.37%	25.77%	<0.05
MDW	21.39 ± 5.33	21.10 ± 5.46	<0.05
NLR	10.34 ± 11.49	10.62 ± 9.93	0.29
WBC (10^3/μL)	11.85 ± 6.94	11.43 ± 20.03	0.41
CRP (mg/dL)	7.12 ± 8.22	8.82 ± 10.94	<0.05
Bacteremia	8.02%	5.65%	<0.05

Abbreviations: DM, diabetes mellitus; CVD, cardiovascular disease; CKD, chronic kidney disease; SBP, systolic blood pressure; DBP, diastolic blood pressure; SpO_2, peripheral oxygen saturation; GCS, Glasgow Coma Scale; qSOFA, quick Sequential (Sepsis-related) Organ Failure Assessment; MDW, monocyte distribution width; NLR, neutrophil-to-lymphocyte ratio; WBC, white blood cell count; CRP, C-reactive protein.

Table S2. Most frequently isolated Gram-negative and Gram-positive pathogens from blood cultures

Group	Rank	Pathogen	n	%
**Gram-negative**	1	*Escherichia coli*	771	29.8
	2	*Klebsiella pneumoniae*	302	11.7
	3	*Proteus mirabilis*	89	3.4
	4	*Pseudomonas aeruginosa*	51	2.0
	5	*Salmonella* group D (non-typhi)	32	1.2
**Gram-positive**	1	*Staphylococcus aureus*	214	8.3
	2	*Enterococcus faecalis*	57	2.2
	3	*Streptococcus agalactiae*	55	2.1
	4	*Streptococcus anginosus*	36	1.4
	5	*Streptococcus dysgalactiae*	27	1.0

Footnotes:

a Percentages were calculated using the total number of positive blood culture isolates as the denominator.

b n denotes the number of isolates (not unique patients).

Table S3. Multivariable logistic regression analysis for bacteremia adjusted for medical history, initial vital signs, and laboratory biomarkers

Variable	Model 1 aOR (95% CI)	p value	Model 2 aOR (95% CI)	p value
**Age**	1.017 (1.014–1.021)	<0.05	1.017 (1.013–1.021)	<0.05
**Sex**	0.984 (0.877–1.105)	0.79	1.040 (0.921–1.174)	0.53
**Hypertension**	1.087 (0.952–1.242)	0.22	1.010 (0.879–1.161)	0.89
**DM**	1.385 (1.212–1.584)	<0.05	1.328 (1.154–1.528)	<0.05
**CVD**	1.061 (0.908–1.239)	0.46	1.087 (0.923–1.281)	0.32
**Pulmonary Disease**	0.518 (0.260–1.033)	0.06	0.490 (0.242–1.210)	0.08
**Liver Disease**	1.622 (0.974–2.700)	0.06	2.180 (0.987–3.692)	0.06
**Stroke**	1.399 (1.124–1.741)	<0.05	1.526 (1.216–1.916)	<0.05
**Neoplasm**	0.817 (0.420–1.588)	0.55	0.789 (0.397–1.570)	0.51
**CKD**	1.072 (0.807–1.423)	0.63	1.069 (0.794–1.439)	0.66
**Body temperature (°C)**	1.406 (1.337–1.479)	<0.05	1.434 (1.359–1.513)	<0.05
**Heart rate (bpm)**	1.028 (1.021–1.35)	<0.05	1.000 (0.998–1.003)	0.24
**Respiratory rate**	0.962 (0.942–0.982)	<0.05	0.962 (0.941–0.983)	<0.05
**SBP**	1.009 (1.006–1.012)	<0.05	1.006 (1.003–1.009)	<0.05
**SpO_2 (%)**	1.007 (0.994–1.019)	0.29	1.012 (0.999–1.025)	0.08
**GCS**	1.000 (0.977–1.023)	0.99	1.004 (0.979–1.028)	0.78
**MDW**	1.132 (1.122–1.142)	<0.05	1.095 (1.084–1.106)	<0.05
**WBC**	—	—	1.008 (1.001–1.015)	<0.05
**NLR**	—	—	1.024 (1.021–1.028)	<0.05
**CRP**	—	—	1.042 (1.035–1.048)	<0.05

Adjusted odds ratios (aORs) with 95% confidence intervals (CIs) were calculated using multivariable logistic regression models to identify factors independently associated with bacteremia. Model 1 was adjusted for patient demographics, comorbidities, and initial vital signs at emergency department presentation. Model 2 additionally included laboratory biomarkers. Continuous variables were analyzed per unit increase. Abbreviations: aOR, adjusted odds ratio; CI, confidence interval; DM, diabetes mellitus; CVD, cardiovascular disease; CKD, chronic kidney disease; SBP, systolic blood pressure; SpO_2, peripheral oxygen saturation; GCS, Glasgow Coma Scale; MDW, monocyte distribution width; WBC, white blood cell count; NLR, neutrophil-to-lymphocyte ratio; CRP, C-reactive protein.

Table S4. Logistic regression coefficients (β) / odds ratios (OR) with 95% CIs and final equations for the two-biomarker models.

Model	Equation	Term	OR (95% CI)	β (ln[OR])
MDW + CRP	logit(P) = −5.522 + 0.107·MDW + 0.049·CRP	Intercept	0.004 (0.004–0.006)	−5.522
		MDW	1.113 (1.103–1.123)	0.107
		CRP	1.050 (1.044–1.057)	0.049
MDW + WBC	logit(P) = −5.809 + 0.135·MDW + 0.028·WBC	Intercept	0.003 (0.002–0.003)	−5.809
		MDW	1.145 (1.136–1.155)	0.135
		WBC	1.028 (1.020–1.035)	0.028
MDW + NLR	logit(P) = −5.809 + 0.132·MDW + 0.030·NLR	Intercept	0.003 (0.002–0.003)	−5.809
		MDW	1.141 (1.131–1.150)	0.132
		NLR	1.030 (1.026–1.033)	0.030

Predicted probability equation: P(bacteremia) = 1 / [1 + exp{−(β0 + β1 × MDW + β2 × X)} (where X = CRP, WBC, or NLR depending on the model.); Abbreviations: MDW, monocyte distribution width; WBC, white blood cell count; CRP, C-reactive protein; NLR, neutrophil-to-lymphocyte ratio; OR, odds ratio; CI, confidence interval; β, regression coefficient; logit(P), log-odds of bacteremia; exp, exponential function; P(bacteremia), predicted probability of bacteremia.
